# A Novel Approach to Assessment of Perceptual-Motor Efficiency and Training-Induced Improvement in the Performance Capabilities of Elite Athletes

**DOI:** 10.3389/fspor.2021.729729

**Published:** 2021-10-01

**Authors:** Gary B. Wilkerson, Dustin C. Nabhan, Tyler S. Perry

**Affiliations:** ^1^Department of Health and Human Performance, University of Tennessee at Chattanooga, Chattanooga, TN, United States; ^2^Oslo Sports Trauma Research Center, Norwegian School of Sport Science, Oslo, Norway; ^3^Orthopedics and Sports Medicine, Emory Healthcare, Atlanta, GA, United States

**Keywords:** sport-related concussion, mild traumatic brain injury, reactive agility, musculoskeletal injury, intra-individual variability, asymmetry, neuromechanics

## Abstract

Standard clinical assessments of mild traumatic brain injury are inadequate to detect subtle abnormalities that can be revealed by sophisticated diagnostic technology. An association has been observed between sport-related concussion (SRC) and subsequent musculoskeletal injury, but the underlying neurophysiological mechanism is not currently understood. A cohort of 16 elite athletes (10 male, 6 female), which included nine individuals who reported a history of SRC (5 male, 4 female) that occurred between 4 months and 8 years earlier, volunteered to participate in a 12-session program for assessment and training of perceptual-motor efficiency. Performance metrics derived from single- and dual-task whole-body lateral and diagonal reactive movements to virtual reality targets in left and right directions were analyzed separately and combined in various ways to create composite representations of global function. Intra-individual variability across performance domains demonstrated very good SRC history classification accuracy for the earliest 3-session phase of the program (Reaction Time Dispersion AUC = 0.841; Deceleration Dispersion AUC = 0.810; Reaction Time Discrepancy AUC = 0.825, Deceleration Discrepancy AUC = 0.794). Good earliest phase discrimination was also found for Composite Asymmetry between left and right movement directions (AUC = 0.778) and Excursion Average distance beyond the minimal body displacement necessary for virtual target deactivation (AUC = 0.730). Sensitivity derived from Youden's Index for the 6 global factors ranged from 67 to 89% and an identical specificity value of 86% for all of them. Median values demonstrated substantial improvement from the first 3-session phase to the last 3-session phase for Composite Asymmetry and Excursion Average. The results suggest that a Composite Asymmetry value ≥ 0.15 and an Excursion Average value ≥ 7 m, provide reasonable qualitative approximations for clinical identification of suboptimal perceptual-motor performance. Despite acknowledged study limitations, the findings support a hypothesized relationship between whole-body reactive agility performance and functional connectivity among brain networks subserving sensory perception, cognitive decision-making, and motor execution. A complex systems approach appears to perform better than traditional data analysis methods for detection of subtle perceptual-motor impairment, which has the potential to advance both clinical management of SRC and training for performance enhancement.

## Introduction

At all ages and levels of competitive sports, athletes are exposed to the potential for injuries that can result in performance impairment (Bahr, 2014), progressive disability (Maffulli et al., 2010), and reduced health-related quality of life (Filbay et al., 2019). Sport-related concussion (SRC) specifically refers to a single head impact that produces symptoms of a mild traumatic brain injury (mTBI), but the cumulative effects of multiple head impacts that do not elicit symptoms may ultimately produce similar long-term effects (Montenigro et al., 2017; Hirad et al., 2019; Hunter et al., 2019). Numerous recent studies have documented a substantial increase in musculoskeletal injury incidence following SRC occurrence (McPherson et al., 2018), but the neurophysiological mechanism that elevates such risk is not understood (Brown et al., 2015; Lynall et al., 2017; Howell et al., 2018; Buckley et al., 2020). Additionally, the likelihood for a subsequent SRC is great (Brett et al., 2018, 2020), and a history of multiple SRCs appears to further elevate risk for musculoskeletal injury (Houston et al., 2018; Harada et al., 2019). Current clinical guidelines for return to sport following SRC rely on self-reported symptom resolution (Baker and Cinelli, 2014; Harmon et al., 2019), but an asymptomatic neuroinflammatory response may persist for months or years beyond the point of return to normal activity (Ezza and Khadrawyb, 2014; Tremblay et al., 2014; Churchill et al., 2017; Shahim et al., 2017; Yanuck, 2019; Brett et al., 2020). Improved clinical assessment methods are needed to identify subtle alterations in brain function that could elevate risk for musculoskeletal injury, occurrence of a subsequent SRC, or development of a neurodegenerative disorder.

Relatively recent technological advances in neuroimaging and neurophysiological testing have generated a dramatic increase in understanding of neural information processing by brain networks (Bressler and Menon, 2010; van den Heuvel and Pol, 2010). Regardless of measurement method, temporal variability derived from a combination of excitatory and inhibitory neural signals appears to be an inherent property of brain function (Garrett et al., 2013). The term *heterostasis* refers to the phenomenon of variability in one neural system producing consistency in the output of a related system (Williams et al., 2016). Conversely, behavioral performance variability appears to result from unstable flow of electrochemical signals across damaged white matter tracts in the superior longitudinal fasciculus and corpus callosum (Fjell et al., 2011). Brain *executive function* is an inclusive term that refers to the set of neural processes that support goal-directed behaviors, which includes the core processes of working memory, inhibitory control, and cognitive flexibility (Diamond, 2013). Rapid and flexible responses to uncertainty in a changing environment reflects an ability to switch between brain states, which appears to depend on variability in global synchronization of neural activity (Garrett et al., 2013; Hellyer et al., 2015; Deco et al., 2017; Grady and Garrett, 2018).

### Assessment of Perceptual-Motor Efficiency

Impaired information processing efficiency following SRC may be a clinically measurable factor that is highly relevant to future injury risk (Fino et al., 2017), as well as long-term brain health. The term *neuromechanics* has been defined as “the interaction between the nervous system and the mechanical properties of the body” (Enoka, 2002), and the term *neuromechanical responsiveness* has been used to refer to the ability to optimally integrate neurocognitive and neuromuscular processes to generate forces that will meet the demands of rapidly changing environmental challenges (Wilkerson et al., 2017, 2018, 2020). Separate brain networks have been identified that process information specific to visual attention, cognition, and motor functions in a somewhat independent manner, but the respective processes are integrated within specific areas of the thalamus and basal ganglia (Greene et al., 2020). Although sensory perception, cognitive decision-making, and motor execution are often viewed as distinct processes, references to perceptual-cognitive function (Wang et al., 2017; Hadlow et al., 2018; Wilke et al., 2020; Cardoso Fd et al., 2021) and cognitive-motor function (Brown et al., 2015; Hurtubise et al., 2016; Leone et al., 2017) reflect the high degree of interrelated neural processing that is necessary for successful performance of goal-directed actions. Perception involves awareness of sensory inputs and their inferred causes (Adams et al., 2013), and motor activation occurs before a decision-making process has been completed (Selen et al., 2012; Gallivan, 2014). Brain *metastability* refers to the capacity for rapid and flexible integration of information across large-scale networks, which may be differentially affected by the specific locations of microstructural damage induced by a traumatic brain injury (Hellyer et al., 2015). We use the term *perceptual-motor efficiency* to refer to optimal processing of neural information that can be quantified by neuromechanical responsiveness to environmental stimuli (Wilkerson et al., 2021a).

Reaction time (RT) provides a unique behavioral measure that has been directly related to connectivity between key brain networks (Erickson et al., 2005; Niogi S. et al., 2008; Mennes et al., 2011; Jilka et al., 2014; Churchill et al., 2021; Urban et al., 2021), which validates the use of RT as an index of brain information processing efficiency (Jensen, 2006; Marmolejo-Ramos et al., 2015). The nature of the presented stimulus and the required response for a given testing procedure can produce an extremely wide range of RT values among different populations, which can range from ~150 ms for initiation of a horizontal eye saccade in response to a visual stimulus among healthy young adults (Danna-Dos-Santos et al., 2018) to more than 800 ms for a stimulus-response compatibility button-press task among older adults (McAuley et al., 2006). Simple RT refers to speed of response to appearance of a stimulus that does not change, whereas Choice RT requires a decision about whether or not to respond to binary stimuli (i.e., go vs. no-go decision). Discrimination RT involves cognitive interpretation of complex stimuli that determine a correct vs. incorrect response, which prolongs the process of completing the proper response (Jensen, 2006). The Eriksen “flanker test” imposes a visual-cognitive processing demand for interference control that has been recognized as “one of the most important experimental tasks in the history of cognitive psychology” (Ridderinkhof et al., 2021). The task involves determination of the direction indicated by a central arrow that is flanked by pairs of arrows that are either congruent (i.e., <<<<< or >>>>>) or incongruent (<<><< or >><>>). The ability to restrain incorrect responses to the distraction imposed by incongruent flanking arrows provides a measure of executive function (Themanson and Rosen, 2015) that has been related to various aspects of brain structure and function with functional magnetic resonance imaging (Erickson et al., 2005; Fan et al., 2005; Kelly et al., 2008; Mennes et al., 2010, 2011; Zhu et al., 2010), diffusion tensor imaging (Niogi S. et al., 2008; Niogi S. N. et al., 2008; FitzGerald and Crosson, 2011; Fjell et al., 2011), and electroencephalography (De Beaumont et al., 2009; Pontifex et al., 2009; Moore et al., 2014, 2015; Parks et al., 2015; Themanson and Rosen, 2015; Wang et al., 2017; Guth et al., 2018).

Testing of RT has often been done as a single-task assessment that requires a relatively simple motor response (e.g., keyboard tap, mouse click, or button press), which appears to have limited value for identification of a performance deficiency following SRC (Urban et al., 2021). Functional RT assessment involves a whole-body response to visual or auditory stimulus during dynamic activities, such as walking gait, jump landing, single-leg hopping, anticipated cutting, and unanticipated cutting (Lempke et al., 2020; Lynall et al., 2021). Lack of strong correlation between functional RT measures with RT measures derived from a simple motor response in a stationary position suggests that the latter may have lesser relevance to performance of sport-specific movement patterns (Lempke et al., 2020). Whole-body reactive agility (WBRA) involves rapid changes of movement velocity or direction in response to a stimulus (Sheppard et al., 2006). If the nature of a test stimulus does not require a substantial amount of cognitive effort to produce a rapid whole-body movement response, a measure of RT derived from such a test could be described as a representation of WBRA Simple RT. Other neuromechanical performance measures derived from initiation of a stimulus response to whole-body displacement over a specified distance could include speed, acceleration and deceleration (Wilkerson et al., 2018, 2020, 2021a).

Dual-task assessment that requires decision-making during performance of whole-body movements may provide an optimal means to quantify subtle neuromechanical performance deficiencies (Baker and Cinelli, 2014; Brown et al., 2015; Leone et al., 2017; Howell et al., 2018; Kung et al., 2020; Urban et al., 2021). The imposition of simultaneous cognitive and motor demands of sufficient complexity may challenge finite neural resources beyond a point that allows compensatory mechanisms to sustain a given level of single-task performance. Dual-task WBRA can be decomposed into two somewhat distinct tasks: (1) stimulus perception and cognitive interpretation, and (2) generation of a rapid motor response that changes the velocity or direction of body movement (McGinnis et al., 2017). Because finite neural resources require selective prioritization of visual inputs that are most relevant to a behavioral goal (Buschman and Kastner, 2015), identification of a subtle impairment of perceptual-cognitive function requires a task that will challenge detection of salient stimuli (Churchill et al., 2021). The incongruent stimuli of the flanker test impose such a demand for focused attention, as well as a requirement for resolution of stimulus-response conflict that prolongs neural processing time (Moore et al., 2015; Servant and Logan, 2019). In terms of dual-task motor response, bradykinesia (i.e., slowing of movement) has been documented among individuals with a history of SRC (Ozolins et al., 2016; Fueger and Huddleston, 2018). Thus, a dual-task WBRA assessment that incorporates the flanker test appears to provide a good method for quantification of perceptual-motor efficiency (Supplementary Figure).

### Potential for Improved Risk Screening and Individualized Interventions

Considering the differing specialized functions of the two brain hemispheres (Serrien et al., 2006; Takeuchi et al., 2012), along with documented microstructural disruption within the connecting white matter tracts following SRC (Niogi S. et al., 2008; Fjell et al., 2011; Womack et al., 2017; Yin et al., 2019), asymmetrical bradykinesia could be an indirect indicator of a subtle impairment of brain function. The majority of studies included in a recent systematic review of literature provided some amount of evidence that lower extremity asymmetry contributes to injury risk, which could be due to a constraint on an athlete's repertoire of movement options (Helme et al., 2021). Previous research that has utilized a Simple WBRA testing protocol has documented greater movement direction performance asymmetries among elite athletes who self-reported a history of SRC compared to those who denied ever having sustained such an injury (Wilkerson et al., 2018, 2020, 2021a). A key factor that differentiates WBRA asymmetry from prior limb performance asymmetry research is the simultaneous engagement of both extremities in generation of body displacements in a given direction (Wilkerson et al., 2021a). The collective research results derived from multiple small-scale projects suggest that poor perceptual-motor performance prospectively associates with musculoskeletal injury occurrences among college football players (Wilkerson et al., 2017), which raises a question about the potential for injury prevention through reduction of performance asymmetries (e.g., RT, speed, acceleration, or deceleration).

Appropriate screening test design, along with proper interpretation of results, offers the potential to identify individuals who would derive greatest benefit from targeted interventions that can reduce risk for a future adverse outcome (Hirad et al., 2019; Stern et al., 2020). However, some experts consider the majority of available evidence inadequate to support the use of sport injury risk screening results in making clinical decisions (Hegedus and Cook, 2015; Bahr, 2016). Other experts believe reductionist study designs and data analyses should be replaced with a complex systems approach for development of injury prevention strategies (Quatman et al., 2009; Bittencourt et al., 2016; Kenzie et al., 2017; Fonseca et al., 2020). A reductionist approach involves a search for isolated risk factors that cause injury (Bittencourt et al., 2016), whereas a complex systems approach involves a search for high-order variables that represent the collective function of multiple interacting system elements that lead to the emergence of injury susceptibility (Fonseca et al., 2020). For example, a *Composite Asymmetry* value derived from multiple WBRA performance metrics has been shown to provide better SRC history discrimination than any single asymmetry metric (Wilkerson et al., 2020, 2021a). *Excursion* (i.e., body displacement beyond that required to reach a target) represents a possible cumulative indicator of suboptimal sensorimotor integration. Other composite performance variables, such as a *Global Index* derived from multiple standardized test scores, may provide a sufficiently sensitive means to identify a subtle deficiency in neural processing efficiency (Roalf et al., 2016).

An extensive body of literature pertaining to change in cognitive performance attributable to the aging process and neurodegenerative conditions has documented greater value of intra-individual (i.e., within-person) variability measures than between-group comparisons of mean values for early detection of impairment (Costa et al., 2019). Intra-individual variability (IIV) is believed to reflect endogenous and exogenous influences on the efficiency of executive control processes, rather than being due to random error or lack of measurement reliability (MacDonald et al., 2009). Different types of IIV include the following: (1) *Inconsistency*, which can refer to either trial-to-trial variability during a single measurement session or variability in performance across measurement sessions conducted on different days (Holtzer et al., 2008; MacDonald et al., 2009; Costa et al., 2019), (2) *Dispersion*, which refers to variability in an individual's standardized scores across multiple performance domains (Holtzer et al., 2008; MacDonald et al., 2009; Roalf et al., 2016; Costa et al., 2019), and (3) *Discrepancy*, which refers to the difference between the maximum and minimum standardized scores from a set of measures representing multiple performance domains (Schretlen et al., 2003). Because neural processing efficiency may be affected by multiple transient factors that are difficult to control (e.g., motivation, fatigue, stress, emotions, or pain), measurements acquired over multiple days are likely to provide a better performance index than measurements acquired on a single occasion Furthermore, transient exogenous factors may have differential effect magnitudes on the neural processing efficiency of individuals who experience persisting adverse effects from a prior SRC.

Multicomponent training for improved movement control has been shown to reduce the incidence of both musculoskeletal injury and SRC among rugby players (Hislop et al., 2017; Attwood et al., 2018), and we have documented improvement of WBRA performance following upper extremity perceptual-motor training among elite athletes who self-reported a history of SRC (Wilkerson et al., 2021a). Thus, the overall goal of this study was to develop a method for accurate identification of athletes who may possess a subtle and potentially modifiable impairment in perceptual-motor processing efficiency, which might be used to guide training for simultaneously enhancement of sport performance capabilities and reduction of risk for future injury. Specifically, the purposes of this exploratory study included: (1) Assessment of the reliability of various WBRA performance metrics across the entire training period, (2) Assessment of the discriminatory power of each WBRA performance metric for identification of athletes who self-reported a history of SRC, (3) Identification of any differences in WBRA performance metrics between early and late phases of training, including any effect attributable to self-reported history of SRC, and (4) Comparison of the classification accuracy provided by Composite Asymmetry and Excursion values to that of Dispersion and Discrepancy values derived from different WBRA performance metrics.

## Materials and Methods

A cohort of 16 elite athletes at a residential training center volunteered to participate in a 12-session WBRA training program (Table 1). Any athlete receiving treatment for any acute injury or illness was excluded from participation. The Institutional Review Board of the University of Tennessee at Chattanooga approved all study procedures. Surveys administered prior to initiation of the first training session included the Sports Fitness Index (Wilkerson et al., 2016), the Overall Wellness Index (Wilkerson et al., 2021b), and the Depression, Anxiety, and Stress Scale (Edmed and Sullivan, 2012), which were used to confirm greater frequency of self-reported problems among the athletes who affirmed a history of at least one SRC (HxSRC).

**Table 1:** Cohort characteristics; height and mass: mean ± standard deviation; age and survey scores: median (range).

	**History of sport-related concussion**		**No history of sport-related concussion**	
*N*	9		7	
Age (years)	25 (21–44)		20 (19–30)	
Sex	Male	Female	Male	Female
	5 (56%)	4 (44%)	5 (71%)	2 (29%)
Height (cm)	168.7 ± 8.3	157.5 ± 3.7	177.7 ± 8.3	165.7 ± 0.0
Mass (kg)	65.6 ± 8.7	58.3 ± 6.9	80.2 ± 11.2	80.1 ± 9.8
Sport:				
Figure skating	1		1	
Gymnastics	2		0	
Marathon	1		0	
Shooting	0		5	
Wrestling	5		1	
Sport Fitness Index (0–100)	50 (40–72)		76 (44–88)	
Overall Wellness Index (0–100)	60 (28–86)		84 (48–94)s	
DASS-21^*^ (63-0)	12 (31-4)		9 (18-0)	

* *Depression, Anxiety, and Stress Scale (21-Item Version; low core represents optimal status)*.

### Assessment and Training Procedures

Each athlete was provided with verbal instructions and the opportunity to perform each of 4 different WBRA task modes during a familiarization session that preceded the first training session. Each training session involved completion of 8 movement sequences (i.e., repetitions) for each of 4 WBRA task modes, which required a total of only 3–4 min per session. To accommodate differing schedules for sport-specific activities, the time intervals between sessions were not identical for each participant. Each athlete completed 2–4 WBRA training sessions per week for a total of 12 sessions that were each separated by a period of 24–72 h.

**Figure 1:**
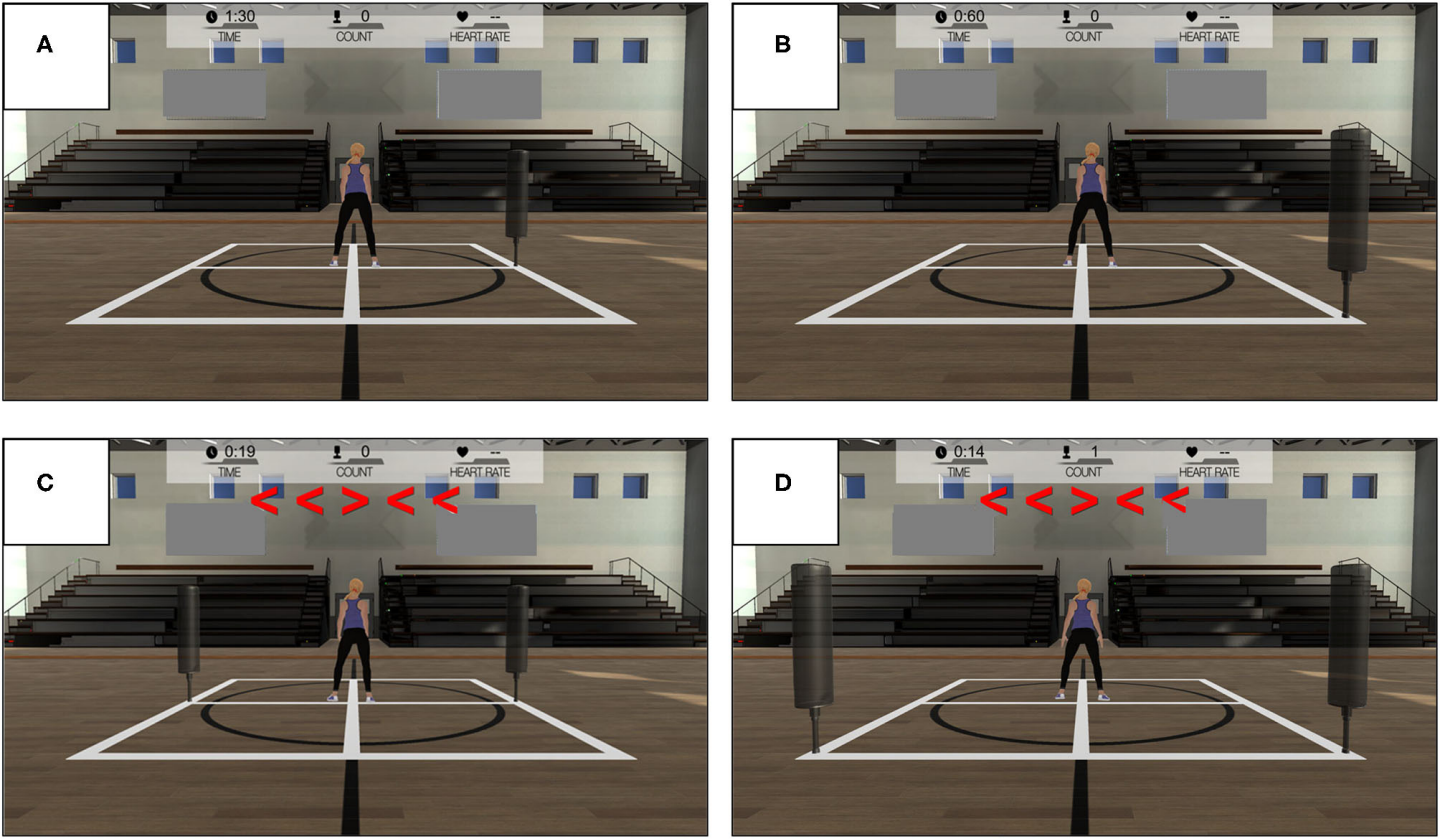
Whole-body reactive agility test modes: **(A)** Lateral Single-Task, **(B)** Diagonal Single-Task, **(C)** Lateral Dual-Task, and **(D)** Diagonal Dual-Task.

The WBRA task modes involved either *Lateral* or *Diagonal* whole-body movements in response to visual targets displayed on a 48 X 86 cm monitor located 1.83 m in front of the athlete. The system used to administer the task (TRAZER® Sports Stimulator, Traq Global Ltd; Westlake, OH) has been shown to provide good test-retest measurement reliability and a valid representation of movement precision in deactivation of virtual targets (Hogg et al., 2021). From a central starting position, each reactive movement in a left or right direction was performed with the goal to quickly deactivate a virtual target on the monitor by moving the body core to a corresponding 2-dimensional X-Y spatial coordinate within the performance space (Figure 1). Immediately after virtual target deactivation, the athlete reversed the movement direction to rapidly return to the original central position for the start of another trial. A single-task (*ST*) mode presented a single target on either the left side (4 repetitions) or right side (4 repetitions) of the monitor, which served as a simple visual cue for a reactive movement in the correct direction. Lateral movement, direction reversal, and return to the central position required a minimum body displacement of 1.83 m for each of 4 left and 4 right targets presented in a random order. Diagonal movement, direction reversal, and return to the central position required a minimum body displacement of 2.59 m for each of 4 back-left and 4 back-right targets presented in random order. A dual-task (*DT*) mode simultaneously presented targets on both the left and right sides of the monitor, with a central display of flanker test arrows in either a congruent configuration (4 repetitions) or incongruent configuration (4 repetitions) for 500 ms. The direction indicated by the center arrow of the flanker test display served as the cue for the movement direction in a randomly determined left or right direction, which added a cognitive challenge to the task (Supplementary Materials). This study used an early version of the DT mode, which required 25% greater lateral displacement and 13% greater diagonal displacement than the ST task mode.

### Performance Metrics

*Reaction Time* was defined as the average amount of time that elapsed between visual target appearance on the monitor and 20 cm of body core displacement in the correct movement direction for the 8 repetitions of the Lateral-ST and Diagonal-ST task modes. The Lateral-DT and Diagonal-DT task modes defined RT as the time that elapsed between the appearance of a flanker test arrow set on the monitor and 15 cm of body core displacement in the correct movement direction. Other WBRA performance metrics, including *Speed, Acceleration*, and *Deceleration*, were derived from the average of body core displacement values for each movement sequence (i.e., central starting position to target and back to central starting position). With the exception of *Excursion* (i.e., total distance covered in excess of the minimum distance required to deactivate 8 targets for a given task mode), the motion analysis system provided average performance values for left vs. right movement directions. Asymmetry for RT, Speed, Acceleration, and Deceleration was defined as the ratio of the absolute difference between performance values for the two directions to the better of the two performance values.

### Data Analyses

Assessment of any group differences in survey scores utilized non-parametric Mann-Whitney tests. Analysis of the WBRA data was initially focused on basic time and distance performance metrics derived from each of the four task modes. Procedures corresponding to the 4 purposes of the study were as follows:

#### Measurement Reliability

The intraclass correlation coefficient (ICC) was calculated using a 2-way mixed-effects and average of measures (3, K) method for consistency across the 12 training sessions. Reliability values were calculated separately for left and right movement directions, the *Average* of left and right movement directions, and *Asymmetry* in left and right movement directions. Qualitative interpretation of ICC_3,K_ values were as follows: <0.50 poor; 0.50–0.74 moderate; 0.75–0.89 good; ≥0.90 excellent (Lynall et al., 2021).

#### Discriminatory Power

Classification accuracy for differentiation of HxSRC vs. no self-report of prior SRC (NoSRC) was represented by a receiver operating characteristic area under curve (AUC) value for each of 4 phases of the 12-session training program (i.e., Phase 1: sessions 1–3; Phase 2: sessions 4–6; Phase 3: sessions 7–9; Phase 4: sessions 10–12). Qualitative interpretation of the AUC value derived from the 3-session average for a given performance metric were as follows: <0.60 poor discrimination; 0.60–0.74 possibly helpful discrimination; ≥0.75 clearly useful discrimination (Hosmer and Lemeshow, 2000; Alba et al., 2017).

#### Pre- to Post-training Change

Parametric analysis of the WBRA data used repeated measures analysis of variance (ANOVA) to assess difference between Phase 1 and Phase 4, difference between Groups (i.e., HxSRC and NoSRC), and Phase X Group interaction for each performance metric, as well as Asymmetry for each one that provided direction-specific data. The distribution normality of each variable was assessed by the Shapiro-Wilk test, with *P* < 0.05 deemed non-normal. In such cases, logarithmic, square root, and reciprocal transformations of the data were done to identify the best means to achieve a more normal distribution. Statistical significance was defined as *P* < 0.05 for all test results. Because of the study's exploratory purpose, no adjustment was made for multiple comparisons (Rothman, 1990; Cao and Zhang, 2014).

#### Comparisons of Derived Metrics

To assess IIV, each bilateral performance metric was converted into a standardized t-score (mean = 50; standard deviation = 10) for each athlete. Because a high value for RT represents poor performance, it was multiplied by −1 in the process of mathematical conversion to a t-score. Thus, a t-score > 50 represented superior performance for RT, Speed, Acceleration, and Deceleration. Dispersion was calculated as the standard deviation of 8 t-scores for a given performance metric and training session (i.e., 2 movement directions X 4 task modes). Discrepancy was calculated as the difference between the maximum and minimum t-scores for a given performance metric and training session. Both representations of IIV were averaged over the 3 consecutive sessions that defined a Phase of the training program. Because the motion analysis system did not provide data for each repetition of a given task mode, within-session inconsistency for the performance metrics could not be quantified. However, the Asymmetry metric does capture inconsistency of performance in left vs. right movement directions, which is derived from an average of 4 repetitions in either direction. Thus, Asymmetry for the 4 task modes was averaged over the 3 sessions of a given training Phase for comparison to Dispersion and Discrepancy values. Excursion average for the four task modes was also evaluated as a possible indicator of imprecise visuospatial guidance of whole-body movements.

To assess the discriminatory power of Dispersion, Discrepancy, Asymmetry, and Excursion for identification of HxSRC, and the relative value of each as a potential Global Index of perceptual-motor efficiency, receiver operating characteristic analysis was performed for each of the 4 bilateral performance metrics, a Composite Dispersion value that represented the standard deviation of 16 t-scores (i.e., 2 movement directions × 4 task modes × 4 metrics), a Composite Discrepancy value that represented the difference between the maximum and minimum of the 16 t-scores, a Composite Asymmetry value derived from the average for the 4 bilateral performance metrics, and Excursion average for the 4 test modes. Results that yielded a Phase 1 AUC value > 0.75 were compared to Phase 4 results for the corresponding predictor to assess a possible differential influence of the training program on performance for HxSRC and NoSRC athletes. Logistic regression analysis was used to assess a possible effect of sex on the results for Phase 1 or Phase 4. Youden's Index was used to identify the Phase 1 cut point that provided maximum classification accuracy, which was compared to that for Phase 4. Global values that provided good discrimination were further assessed by non-parametric tests. The Wilcoxon Signed-Ranks test was used to assess Phase 1 to Phase 4 change and the Mann-Whitney test was used to identify any significant Group differences.

## Results

Self-reported HxSRC at 3.0 ± 2.2 years prior to testing (range: 4 months−8 years) represented 56% of the cohort (9/16; 5 males, 4 females). The number of prior SRCs ranged from 1 to 3, with 44% (4/9) reporting a single SRC and 56% (5/9) reporting 2 or more SRCs. All 3 survey scores demonstrated poorer values for HxSRC athletes compared to NoSRC athletes, with the most significant difference found for the Sport Fitness Index (*P* = 0.031). Non-significant differences in survey scores were found for the Overall Wellness Index (*P* = 0.142) and the Depression, Anxiety, and Stress Survey (*P* = 0.408).

The majority of WBRA performance metrics demonstrated excellent reliability for each task mode, whereas Asymmetry ICC values were generally quite poor (Table 2; statistically significant results identified by bold font). Excursion for the Lateral-DT task mode was the only performance metric that demonstrated clearly useful Phase 1 discrimination, which was also potentially useful for the Diagonal-ST and Diagonal-DT task modes (Table 3; statistically significant results identified by bold font). Repeated measures ANOVA failed to identify any statistically significant effects for any performance metric derived from the Lateral-ST or Diagonal-ST task modes (Table 4). Conversely, use of the flanker test to specify the correct WBRA movement direction resulted in statistically significant change from Phase 1 to Phase 4 for 3 Lateral-DT performance metrics and all 5 of the performance metrics for the Diagonal-DT task mode (Table 5; statistically significant *P*-values identified by bold font). Excursion for the Lateral-DT task mode was the only performance metric that demonstrated a significant Phase X Group interaction effect.

**Table 2:** Intraclass Correlation Coefficient (3, K) values for consistency of measurements across 12 training sessions.

**Mode**	**Metric**	**Left**	**Right**	**Average**	**Asymmetry**
Lateral Single-Task	Reaction Time	0.732	0.810	0.832	0.335
	Speed	**0.968**	**0.964**	**0.968**	0.150
	Acceleration	**0.915**	**0.929**	**0.943**	0.175
	Deceleration	**0.917**	**0.923**	**0.945**	0.315
	Excursion	0.868			
Lateral Dual-Task	Reaction Time	0.732	0.639	0.811	0.128
	Speed	**0.973**	**0.948**	**0.967**	0.385
	Acceleration	**0.932**	**0.935**	**0.956**	^*^
	Deceleration	**0.917**	**0.923**	**0.945**	^*^
	Excursion	0.873			
Diagonal Single-Task	Reaction Time	0.839	0.870	0.851	^*^
	Speed	**0.951**	**0.962**	**0.970**	0.123
	Acceleration	0.599	0.812	**0.910**	0.047
	Deceleration	0.766	0.876	0.889	0.141
	Excursion	0.882			
Diagonal Dual-Task	Reaction Time	0.876	0.878	**0.928**	0.603
	Speed	**0.942**	**0.947**	**0.965**	0.579
	Acceleration	0.871	**0.908**	**0.951**	^*^
	Deceleration	**0.928**	**0.903**	**0.964**	^*^
	Excursion	**0.907**			

* *Value ≤ 0.000. Excellent consistency identified by bold font*.

**Table 3:** Receiver operating characteristic area under curve values for Average^*^ of performance metrics for each task mode.

**Mode**	**Metric**	**Phase 1**	**Phase 2**	**Phase 3**	**Phase 4**
Lateral Single-Task	Reaction Time	0.429	0.349	0.524	0.571
	Speed	0.333	0.476	0.524	0.556
	Acceleration	0.524	0.381	0.429	0.476
	Deceleration	0.349	0.365	0.460	0.492
	Excursion	0.540	0.440	0.508	0.444
Diagonal Single-Task	Reaction Time	0.317	0.571	0.476	0.365
	Speed	0.444	0.492	0.556	0.540
	Acceleration	0.333	0.413	0.365	0.460
	Deceleration	0.270	0.413	0.365	0.460
	Excursion	0.667	0.413	0.492	0.444
Lateral Dual-Task	Reaction Time	0.587	**0.810**	**0.810**	0.667
	Speed	0.397	0.492	0.556	0.508
	Acceleration	0.381	0.381	0.492	0.556
	Deceleration	0.397	0.444	0.476	0.460
	Excursion	**0.810**	0.492	0.603	0.508
Diagonal Dual-Task	Reaction Time	0.667	0.508	0.603	0.587
	Speed	0.413	0.397	0.476	0.508
	Acceleration	0.317	0.302	0.333	0.429
	Deceleration	0.317	0.349	0.286	0.429
	Excursion	0.651	0.556	0.492	0.603

* *Performance values for Left and Right movement directions included in average. Statistically significant results identified by bold font*.

**Table 4:** Group mean ± standard deviation for Single-Task performance.

**Mode**	**Metric**	**Units**	**Group**	**Phase 1**	**Phase 4**	**Change**	***P*_**Ph**_**	***P*_**Gr**_**	***P*_**PhXGr**_**
Lateral Single-Task	Reaction Time	ms	HxSRC	569 ± 107	608 ± 110	−39 ± 95	0.422	0.980	0.626
			NoSRC	585 ± 58	595 ± 130	−10 ± 144			
	Speed	m·s^−1^	HxSRC	1.03 ± 0.18	1.01 ± 0.19	−0.02 ± 0.09	0.386	0.655	0.133
			NoSRC	0.95 ± 0.13	1.02 ± 0.17	0.07 ± 0.13			
	Acceleration	m·s^−2^	HxSRC	3.61 ± 0.95	4.01 ± 1.06	0.45 ± 0.82	0.126	0.978	0.859
			NoSRC	3.66 ± 0.62	4.02 ± 0.92	0.36 ± 1.16			
	Deceleration	m·s^−2^	HxSRC	3.22 ± 0.71	3.13 ± 0.71	−0.09 ± 0.50	0.846	0.493	0.456
			NoSRC	2.90 ± 0.48	3.05 ± 0.67	0.15 ± 0.77			
	Excursion	m	HxSRC	3.61 ± 2.07	2.00 ± 1.77	−1.61 ± 2.13	0.064	0.999	0.566
			NoSRC	3.24 ± 1.45	2.37 ± 2.19	−0.87 ± 2.81			
Diagonal Single-Task	Reaction Time	ms	HxSRC	819 ± 126	836 ± 158	−17 ± 84	0.427	0.559	0.985
			NoSRC	853 ± 79	869 ± 68	−16 ± 78			
	Speed	m·s^−1^	HxSRC	1.03 ± 0.20	1.02 ± 0.19	−0.01 ± 0.10	0.518	0.881	0.283
			NoSRC	1.01 ± 0.16	1.06 ± 0.14	0.05 ± 0.14			
	Acceleration	m·s^−2^	HxSRC	3.33 ± 0.68	3.14 ± 0.70	−0.19 ± 0.82	0.634	0.817	0.295
			NoSRC	2.91 ± 0.63	3.41 ± 1.47	0.49 ± 1.64			
	Deceleration	m·s^−2^	HxSRC	3.00 ± 0.54	3.07 ± 0.69	0.70 ± 0.53	0.204	0.325	0.361
			NoSRC	2.57 ± 0.59	2.98 ± 0.65	0.41 ± 0.90			
	Excursion	m	HxSRC	3.81 ± 2.77	2.36 ± 1.65	−1.46 ± 2.06	0.173	0.614	0.233
			NoSRC	2.70 ± 1.74	2.60 ± 1.36	−0.10 ± 2.28			

*HxSRC, History of Sport-Related Concussion; NoSRC, No History of Sport-Related Concussion; P_Ph_, Phase Difference; P_Gr_, Group Difference; P_PhXGr_, Phase X Group Interaction*.

**Table 5:** Group mean ± standard deviation for Dual-Task performance.

**Mode**	**Metric**	**Units**	**Group**	**Phase 1**	**Phase 4**	**Change**	***P*_**Ph**_**	***P*_**Gr**_**	***P*_**PhXGr**_**
Lateral Dual-Task	Reaction Time	ms	HxSRC	1,157 ± 223	887 ± 84	270 ± 194	**<0.001**	0.328	0.742
			NoSRC	1,074 ± 182	835 ± 113	239 ± 172			
	Speed	m·s^−1^	HxSRC	0.74 ± 0.14	0.79 ± 0.12	0.05 ± 0.12	**0.010**	0.855	0.345
			NoSRC	0.71 ± 0.14	0.81 ± 0.13	0.10 ± 0.07			
	Acceleration	m·s^−2^	HxSRC	3.11 ± 0.71	3.26 ± 0.58	0.15 ± 0.63	0.074	0.629	0.368
			NoSRC	2.83 ± 0.60	3.26 ± 0.92	0.43 ± 0.56			
	Deceleration	m·s^−2^	HxSRC	2.98 ± 0.66	3.21 ± 0.44	0.23 ± 0.61	0.058	0.573	0.665
			NoSRC	2.77 ± 0.57	3.12 ± 0.61	0.35 ± 0.46			
	Excursion	m	HxSRC	10.71 ± 3.41	5.69 ± 2.29	−5.02 ± 2.28	**<0.001**	0.173	**0.017**
			NoSRC	7.57 ± 1.93	5.76 ± 1.11	−1.81 ± 2.42			
Diagonal Dual-Task	Reaction Time	ms	HxSRC	1,050 ± 163	898 ± 91	152 ± 124	**0.001**	0.239	0.145
			NoSRC	950 ± 46	877 ± 91	73 ± 60			
	Speed	m·s^−1^	HxSRC	0.82 ± 0.15	0.87 ± 0.16	0.05 ± 0.11	**0.007**	0.931	0.277
			NoSRC	0.78 ± 0.09	0.89 ± 0.09	0.11 ± 0.09			
	Acceleration	m·s^−2^	HxSRC	3.00 ± 0.64	3.14 ± 0.58	0.14 ± 0.46	**0.017**	0.257	0.177
			NoSRC	2.56 ± 0.35	3.02 ± 0.34	0.46 ± 0.41			
	Deceleration	m·s^−2^	HxSRC	3.11 ± 0.69	3.18 ± 0.72	0.07 ± 0.39	**0.046**	0.259	0.172
			NoSRC	2.65 ± 0.34	2.99 ± 0.38	0.34 ± 0.35			
	Excursion	m	HxSRC	12.15 ± 4.09	7.37 ± 2.99	−4.78 ± 2.91	**<0.001**	0.342	0.524
			NoSRC	10.33 ± 4.79	6.41 ± 1.51	−3.62 ± 4.23			

*HxSRC, History of Sport-Related Concussion; NoSRC, No History of Sport-Related Concussion; P_Ph_, Phase Difference; P_Gr_, Group Difference; P_PhXGr_, Phase X Group Interaction. Statistically significant P-values identified by bold font*.

**Figure 2:**
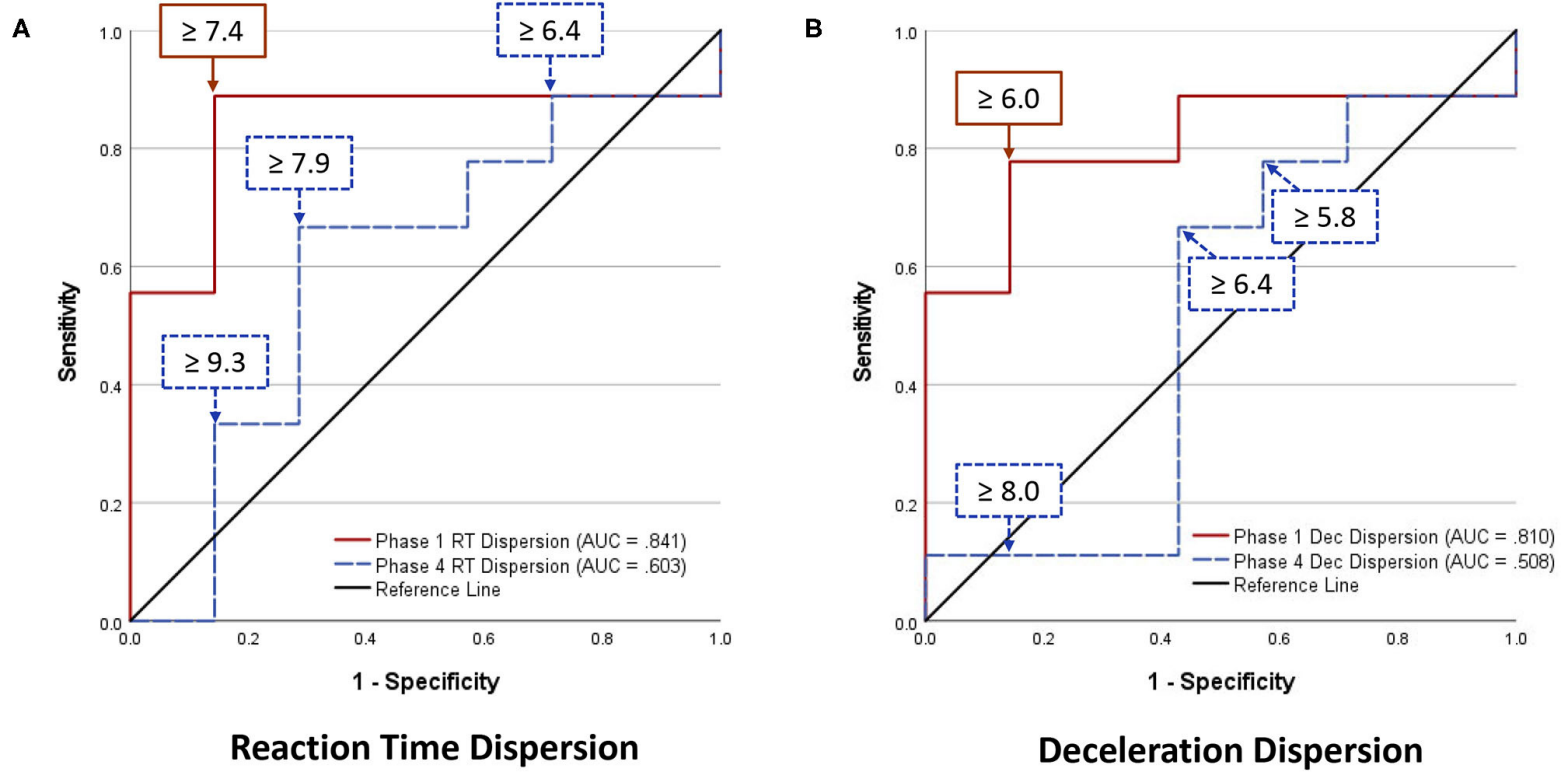
Receiver operating characteristic curves identifying History of Sport-Related Concussion cases in early phase (red solid line; sessions 1–3) and late phase (blue dashed line; sessions 10–12) of 12-session whole-body reactive agility training program on the basis of Dispersion (standard deviation of t-scores for 2 movement directions and 4 task modes): **(A)** Reaction Time Dispersion, **(B)** Deceleration Dispersion.

**Figure 3:**
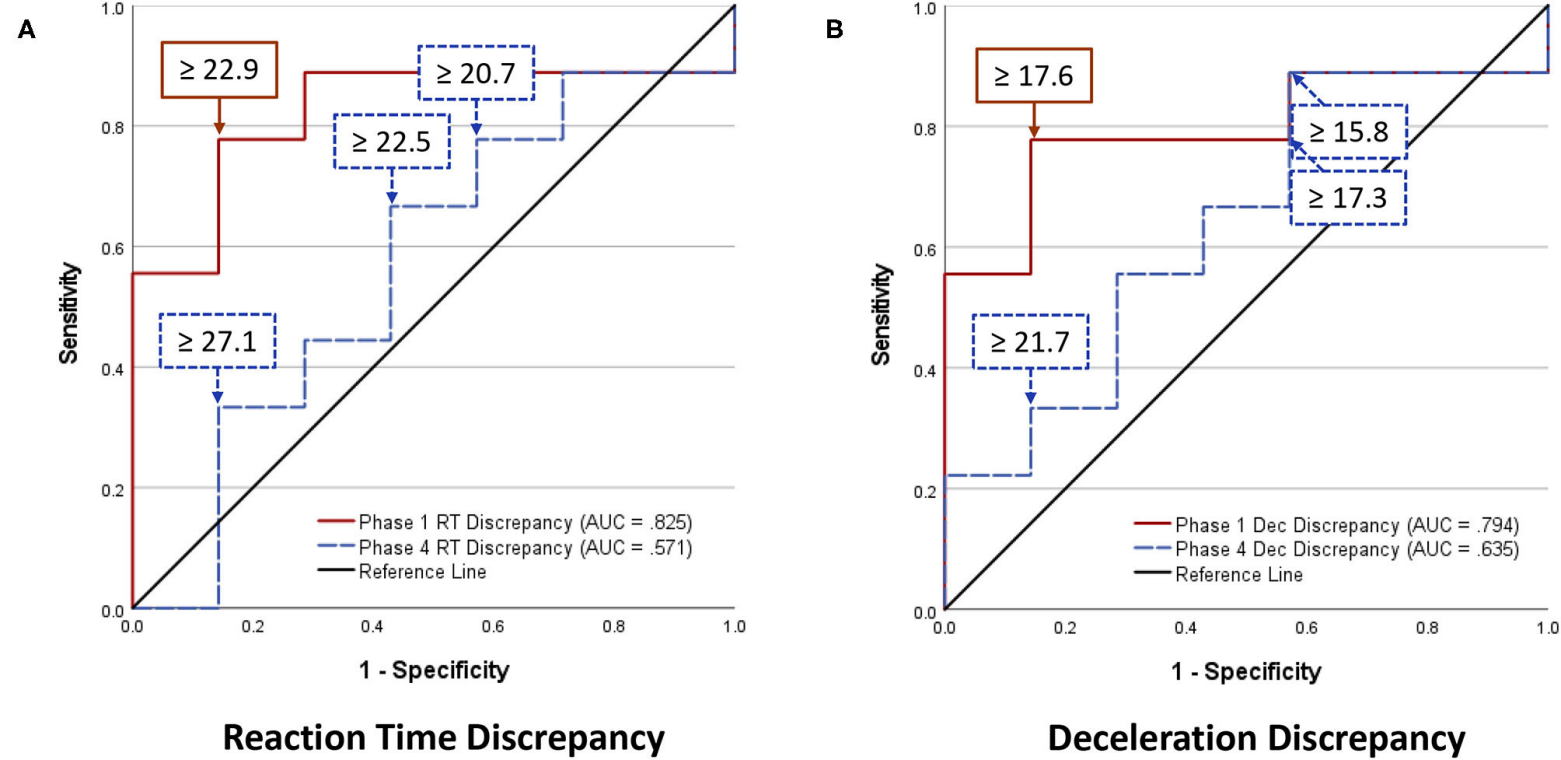
Receiver operating characteristic curves identifying History of Sport-Related Concussion cases in early phase (red solid line; sessions 1–3) and late phase (blue dashed line; sessions 10–12) of 12-session whole-body reactive agility training program on the basis of Discrepancy (maximum–minimum difference) among t-scores for 2 movement directions and 4 task modes: **(A)** Reaction Time Discrepancy, **(B)** Deceleration Discrepancy.

**Figure 4:**
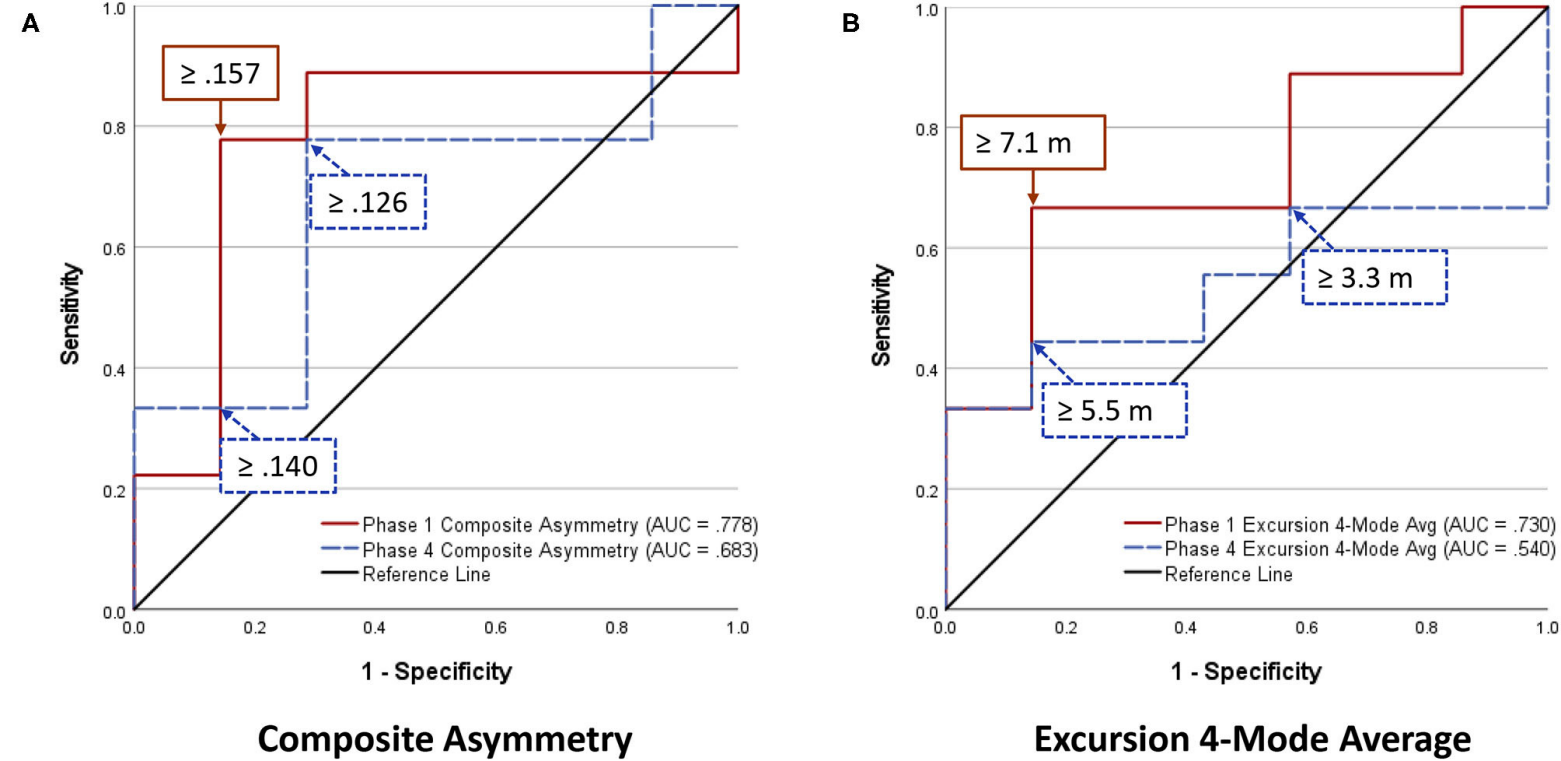
Receiver operating characteristic curves identifying History of Sport-Related Concussion cases in early phase (red solid line; sessions 1–3) and late phase (blue dashed line; sessions 10–12) of 12-session whole-body reactive agility training program: **(A)** Composite Asymmetry (average for 4 task modes and 4 performance metrics), **(B)** Excursion (average for 4 task modes).

Analyses of WBRA Asymmetry in left vs. right movement directions generally demonstrated better Phase 1 discrimination (Table 6; statistically significant P-values identified by bold font) and greater Phase 1 to Phase 4 change for the Composite metric than values specific to RT, Speed, Acceleration, and Deceleration (Tables 7, 8; statistically significant *P-values* identified by bold font). Asymmetry values were generally lower for both the HxSRC and NoSRC groups in Phase 4 compared to Phase 1. A Composite Asymmetry metric representing averaged values across the 4 task modes demonstrated clearly useful Phase 1 discrimination, but IIV measures (i.e., Dispersion and Discrepancy) demonstrated greater levels of Phase 1 discrimination for both RT and Deceleration (Table 9; statistically significant results identified by bold font). A graphic comparison of Phase 1 and Phase 4 discrimination for RT Dispersion and Deceleration Dispersion demonstrates lesser Phase 4 discrimination and poorer Phase 4 values at the same sensitivity level defining optimal Phase 1 discrimination (Figure 2). Similar results were found for RT Discrepancy and Deceleration Discrepancy (Figure 3), as well as Composite Asymmetry and Excursion 4-Mode Average (Figure 4). Despite Composite Asymmetry having demonstrated substantial Phase 4 discrimination, the Youden's index value providing an optimal balance of sensitivity and specificity was substantially smaller than the corresponding Phase 1 value. Sex did not demonstrate a significant effect on any Global Index association with HxSRC. Analysis of median values demonstrated greatest Phase 1 differences between the HxSRC and NoSRC groups for the RT and Deceleration IIV measures (Table 10; statistically significant *P-values* identified by bold font). Greatest Phase 1 to Phase 4 change in median values was evident for Composite Asymmetry and Excursion 4-Mode Average.

**Table 6:** Receiver operating characteristic area under curve values for Asymmetry of Left and Right performance metrics for each task mode.

**Mode**	**Metric**	**Phase 1**	**Phase 2**	**Phase 3**	**Phase 4**
Lateral Single-Task	Reaction Time	0.698	0.476	0.619	0.746
	Speed	0.587	**0.778**	0.595	0.302
	Acceleration	0.476	0.635	0.532	0.675
	Deceleration	0.492	0.317	0.468	0.587
	Composite^*^	**0.794**	0.444	0.667	**0.762**
Diagonal Single-Task	Reaction Time	0.667	0.405	0.500	0.460
	Speed	0.627	0.444	0.698	0.175
	Acceleration	0.476	0.397	0.429	0.373
	Deceleration	0.587	0.690	0.627	0.468
	Composite^*^	0.667	0.460	0.484	0.444
Lateral Dual-Task	Reaction Time	0.270	0.325	0.500	0.524
	Speed	0.537	0.574	0.315	0.713
	Acceleration	0.349	0.714	0.325	0.683
	Deceleration	0.540	0.365	0.619	0.460
	Composite^*^	0.370	0.426	0.426	0.576
Diagonal Dual-Task	Reaction Time	0.563	0.667	0.524	0.397
	Speed	0.310	0.317	0.556	0.476
	Acceleration	0.476	0.635	0.365	0.714
	Deceleration	0.460	0.540	0.683	0.643
	Composite^*^	0.444	0.556	0.429	0.508

* *Average of Asymmetry values for Reaction Time, Speed, Acceleration, and Deceleration. Statistically significant P-values identified by bold font*.

**Table 7:** Group mean ± standard deviation for Single-Task asymmetry.

**Mode**	**Metric**	**Group**	**Phase 1**	**Phase 4**	**Change**	***P*_**Ph**_**	***P*_**Gr**_**	***P*_**PhXGr**_**
Lateral Single-Task	Reaction Time	HxSRC	0.373 ± 0.268	0.203 ± 0.093	0.171 ± 0.342	0.080	**0.008**	0.852
	Asymmetry^a^	NoSRC	0.199 ± 0.095	0.124 ± 0.089	0.074 ± 0.154			
	Speed	HxSRC	0.059 ± 0.023	0.040 ± 0.024	0.019 ± 0.024	0.070	0.353	0.119
	Asymmetry^a^	NoSRC	0.057 ± 0.022	0.054 ± 0.021	0.002 ± 0.033			
	Acceleration	HxSRC	0.116 ± 0.037	0.116 ± 0.037	−0.007 ± 0.052	0.713	0.761	0.842
	Asymmetry	NoSRC	0.107 ± 0.050	0.104 ± 0.066	0.003 ± 0.081			
	Deceleration	HxSRC	0.103 ± 0.057	0.127 ± 0.051	−0.023 ± 0.072	0.630	0.469	0.390
	Asymmetry	NoSRC	0.098 ± 0.034	0.104 ± 0.053	−0.007 ± 0.059			
	Composite	HxSRC	0.161 ± 0.062	0.121 ± 0.024	0.039 ± 0.080	0.126	**0.003**	0.724
	Asymmetry^a^	NoSRC	0.115 ± 0.043	0.097 ± 0.026	0.018 ± 0.055			
Diagonal Single-Task	Reaction Time	HxSRC	0.342 ± 0.246	0.190 ± 0.128	0.152 ± 0.213	**0.025**	0.962	0.155
	Asymmetry^b^	NoSRC	0.216 ± 0.113	0.171 ± 0.056	0.045 ± 0.087			
	Speed	HxSRC	0.117 ± 0.101	0.061 ± 0.029	0.056 ± 0.093	0.416	0.869	0.056
	Asymmetry	NoSRC	0.073 ± 0.031	0.097 ± 0.033	−0.024 ± 0.045			
	Acceleration	HxSRC	0.199 ± 0.088	0.230 ± 0.066	0.004 ± 0.109	0.493	0.705	0.596
	Asymmetry	NoSRC	0.195 ± 0.084	0.227 ± 0.043	−0.031 ± 0.087			
	Deceleration	HxSRC	0.153 ± 0.042	0.124 ± 0.042	0.029 ± 0.060	0.350	0.961	0.661
	Asymmetry	NoSRC	0.143 ± 0.047	0.133 ± 0.057	0.010 ± 0.101			
	Composite	HxSRC	0.203 ± 0.075	0.144 ± 0.041	0.058 ± 0.056	**0.046**	0.458	**0.047**
	Asymmetry	NoSRC	0.157 ± 0.037	0.157 ± 0.027	0.011 ± 0.054			

*HxSRC, History of Sport-Related Concussion; NoSRC, No History of Sport-Related Concussion; P_Ph_, Phase Difference; P_Gr_, Group Difference; P_PhXGr_, Phase X Group Interaction*.

a *Log_10^x^_ transformation of data; means and standard deviations correspond to raw values*.

b *Reciprocal (1/x) transformation of data; means and standard deviations correspond to raw values. Statistically significant P-values identified by bold font*.

**Table 8:** Group mean ± standard deviation for Dual-Task asymmetry.

**Mode**	**Metric**	**Group**	**Phase 1**	**Phase 4**	**Change**	***P*_**Ph**_**	***P*_**Gr**_**	***P*_**PhXGr**_**
Lateral Dual-Task	Reaction Time	HxSRC	0.288 ± 0.242	0.139 ± 0.074	0.149 ± 0.220	**0.002**	0.189	0.150
	Asymmetry	NoSRC	0.497 ± 0.301	0.143 ± 0.124	0.353 ± 0.319			
	Speed	HxSRC	0.092 ± 0.046	0.089 ± 0.041	0.003 ± 0.063	0.431	0.449	0.407
	Asymmetry^a^	NoSRC	0.082 ± 0.042	0.068 ± 0.029	0.014 ± 0.029			
	Acceleration	HxSRC	0.120 ± 0.064	0.103 ± 0.046	0.017 ± 0.033	**<0.001**	0.624	**0.025**
	Asymmetry	NoSRC	0.128 ± 0.012	0.073 ± 0.026	0.055 ± 0.025			
	Deceleration	HxSRC	0.127 ± 0.051	0.112 ± 0.045	0.015 ± 0.072	0.626	0.867	0.798
	Asymmetry	NoSRC	0.124 ± 0.037	0.120 ± 0.069	0.005 ± 0.089			
	Composite	HxSRC	0.157 ± 0.067	0.111 ± 0.029	0.046 ± 0.070	**<0.001**	0.351	0.120
	Asymmetry	NoSRC	0.208 ± 0.077	0.104 ± 0.043	0.107 ± 0.077			
Diagonal Dual-Task	Reaction Time	HxSRC	0.201 ± 0.145	0.172 ± 0.142	0.029 ± 0.134	0.954	0.646	0.704
	Asymmetry	NoSRC	0.163 ± 0.068	0.170 ± 0.044	−0.007 ± 0.067			
	Speed	HxSRC	0.090 ± 0.034	0.085 ± 0.050	0.006 ± 0.061	0.631	0.426	0.952
	Asymmetry	NoSRC	0.102 ± 0.015	0.095 ± 0.038	0.007 ± 0.033			
	Acceleration	HxSRC	0.122 ± 0.068	0.203 ± 0.064	−0.081 ± 0.105	**0.046**	0.248	0.253
	Asymmetry	NoSRC	0.130 ± 0.030	0.154 ± 0.059	−0.042 ± 0.079			
	Deceleration	HxSRC	0.163 ± 0.078	0.168 ± 0.055	−0.004 ± 0.111	0.654	0.832	0.551
	Asymmetry	NoSRC	0.177 ± 0.083	0.146 ± 0.062	0.031 ± 0.120			
	Composite	HxSRC	0.144 ± 0.055	0.157 ± 0.062	−0.013 ± 0.060	0.639	0.862	0.533
	Asymmetry^b^	NoSRC	0.143 ± 0.030	0.141 ± 0.026	0.002 ± 0.041			

*HxSRC, History of Sport-Related Concussion; NoSRC, No History of Sport-Related Concussion; P_Ph_, Phase Difference; P_Gr_, Group Difference; P_PhXGr_, Phase X Group Interaction*.

a *Log_10^x^_ transformation of data; means and standard deviations correspond to raw values*.

b *Reciprocal (1/x) transformation of data; means and standard deviations correspond to raw values. Statistically significant P-values identified by bold font*.

**Table 9:** Receiver operating characteristic area under curve values for intra-individual variability measures (Dispersion and Discrepancy of t-scores for 2 movement directions and 4 task modes), Asymmetry (differences between movement directions averaged for task modes), and Excursion (distances averaged for task modes).

**Global factor**	**Metric**	**Phase 1**	**Phase 2**	**Phase 3**	**Phase 4**
Dispersion	Reaction Time	**0.841**	0.619	0.587	0.603
	Speed	0.683	0.667	0.540	0.476
	Acceleration	0.635	0.635	0.476	0.603
	Deceleration	**0.810**	0.508	0.619	0.508
	Composite^*^	0.651	0.524	0.587	0.524
Discrepancy	Reaction Time	**0.825**	0.619	0.619	0.571
	Speed	0.683	0.683	0.492	0.444
	Acceleration	0.587	0.714	0.508	0.619
	Deceleration	**0.794**	0.508	0.619	0.635
	Composite^*^	0.619	0.534	0.635	0.492
Asymmetry	Reaction Time	0.683	0.349	0.587	0.619
	Speed	0.556	0.452	0.460	0.341
	Acceleration	0.429	0.643	0.413	0.635
	Deceleration	0.500	0.476	0.619	0.595
	Composite^*^	**0.778**	0.397	0.508	0.683
Excursion	4-Mode Average	0.730	0.476	0.540	0.540

* *Composite Dispersion: Average of 16 t-scores for 2 movement directions × 4 task modes × 4 metrics*.

*Composite Discrepancy: Difference between maximum and minimum values for 16 t-scores*.

*Composite Asymmetry: Average of asymmetry values for Reaction Time, Speed, Acceleration, and Deceleration. Statistically significant P-values identified by bold font*.

**Table 10:** Median values for potential high-order indicators of perceptual-motor efficiency.

**Global factor**	**Group**	**Phase 1**	**Phase 4**	***P_***Ph***_***
Reaction Time Dispersion (T-score Standard Deviation)	HxSRC	9.95	8.22	0.139
	NoSRC	7.17	7.76	0.398
	***P***_**Gr**_	**0.023**	0.536	
Deceleration Dispersion (T-score Standard Deviation)	HxSRC	6.99	6.61	0.767
	NoSRC	5.44	6.43	0.128
	***P***_**Gr**_	**0.042**	1.000	
Reaction Time Discrepancy (T-score Max-Min Difference)	HxSRC	27.35	23.10	0.173
	NoSRC	20.65	22.22	0.310
	***P***_**Gr**_	**0.031**	0.681	
Deceleration Discrepancy (T-score Max-Min Difference)	HxSRC	19.89	20.05	0.594
	NoSRC	16.57	18.98	0.128
	***P***_**Gr**_	0.055	0.408	
Composite Asymmetry (4-Metric & 4-Mode Average)	HxSRC	0.172	0.135	**0.008**
	NoSRC	0.152	0.123	**0.018**
	***P***_**Gr**_	0.071	0.252	
Excursion (4-Mode Average; m)	HxSRC	7.20	4.99	**0.011**
	NoSRC	6.20	4.44	0.128
	***P***_**Gr**_	0.142	0.837	

*HxSRC, History of Sport-Related Concussion; NoSRC, No History of Sport-Related Concussion; P_Ph_, Wilcoxon Signed Ranks test result for Phase Difference; P_Gr_, Mann-Whitney test result for Group Difference. Statistically significant P-values identified by bold font*.

## Discussion

### Intra-Individual Variability Association With History of Sport-Related Concussion

Dispersion of standardized scores representing different aspects of neurocognition has been used as a *Global Index* of executive function, which has identified subtle early-stage alteration of neural processing among older adults diagnosed with mild cognitive impairment, dementia, and Alzheimer's disease (Holtzer et al., 2008; MacDonald et al., 2009; Roalf et al., 2016; Costa et al., 2019). Discrepancy between the largest and smallest standardized scores for various measures of cognitive function has been shown to increase with age (Schretlen et al., 2003), which is clearly associated with a decline of mental capabilities that varies among individuals (Wahl et al., 2021). Although utilization of such IIV analysis methods has been advocated for identification of subtle impairment of neural processing in different clinical populations (Costa et al., 2019), our study is the first to demonstrate the potential value of Dispersion and Discrepancy measures for assessment of perceptual-motor efficiency among elite athletes. RT Dispersion demonstrated the greatest HxSRC discriminatory power, with 89% sensitivity and 86% specificity. In terms of clinical utility, a major limitation is the need for normative data from an appropriate reference population to calculate an individual's standardized scores, as well as the need to calculate the standard deviation among them or the difference between largest and smallest values.

Because our Dispersion and Discrepancy measures incorporated neuromechanical performance metrics that were calculated separately for left and right movement directions, some amount of the discriminatory power of these IIV measures probably overlap substantially with that of the Composite Asymmetry metric. Although Excursion did not incorporate separate measures for left and right movement directions, the 4-Mode Average can be viewed as another Global Index of neuromechanical function. In fact, the Phase 1 Youden's Index identified the identical 86% specificity value for RT Dispersion, Deceleration Dispersion, RT Discrepancy, Deceleration Discrepancy, Composite Asymmetry, and Excursion 4-Mode Average. Conversion of Excursion distances (m) to standardized T-scores resulted in a Youden's Index cut point corresponding to an Excursion T-Score 4-Mode Average of 50, with the median for the entire cohort closely approximating a value of 50. The Excursion metric demonstrated good to excellent test-retest reliability for each of the task modes, whereas Asymmetry was highly inconsistent for almost all of the other WBRA performance metrics (Table 2). Despite poor test-retest reliability for Composite Asymmetry, Youden's Index corresponded to 78% sensitivity, which was substantially greater than the 67% sensitivity for Excursion 4-Mode Average. Furthermore, the Composite Asymmetry cut point of ≥0.159 identified by Youden's Index and the median value of 0.157 for the entire cohort strongly support the clinical relevance of a prior finding of Composite Asymmetry ≥0.15 as a potentially good standard for identification of persisting SRC effects on neuromechanical function (Wilkerson et al., 2020). Although clinical utilization of classification cut points for risk factors has been criticized for having poor generalizability (Bahr, 2016), composite values are believed to have greater stability than the individual measurements from which they were derived (Costa et al., 2019; Churchill et al., 2021).

### Neural Basis for Inconsistency of Perceptual-Motor Performance

Inconsistency among successive RT measurements acquired within a relatively short time period represents the most commonly investigated type of IIV. A substantial body of literature identifies RT inconsistency (RTI) as a good indicator of inefficient neural processing, which is believed to primarily depend on attentional control (Jackson et al., 2012; Garrett et al., 2013; Johnson et al., 2015; Wawrzyniak et al., 2016; Grady and Garrett, 2018; Stawski et al., 2019a,b; Shen et al., 2020). The default mode network (DMN) is defined by spontaneous neural activity among widespread areas of the brain mid-line in the absence of external task engagement (Mennes et al., 2010, 2011). Detection of goal-relevant external stimuli by the salience network (SN) simultaneously suppresses DMN activity and activates the ventral attention network (VAN) and the dorsal attention network (DAN), both of which have bidirectional connections with areas in the thalamus, basal ganglia and brainstem that control the rate and locations of saccadic eye movements (Corbetta et al., 2008; Parr and Friston, 2017; Taghdiri et al., 2018). Concurrent SN activation of the executive control network (ECN), which overlaps dorsolateral prefrontal and superior parietal areas of the DAN, exhibits an inverse relationship to the level of DMN activation when attention is externally focused (Seeley et al., 2007; Kelly et al., 2008; Jilka et al., 2014). Key nodes of the SN include the anterior cingulate cortex and the anterior insula, which together play a central role in response inhibition, conflict resolution, and motor planning (Seeley et al., 2007; Kelly et al., 2008; Sridharan et al., 2008; Jilka et al., 2014; Johnson et al., 2015). Numerous studies of RTI have utilized the flanker test to provide a cognitive challenge that requires more extensive brain activation and network integration than simpler visual stimuli (Kelly et al., 2008; Mennes et al., 2010, 2011; Fjell et al., 2011; Parks et al., 2015; Williams et al., 2016; Wang et al., 2017; Olson et al., 2018; McGowan et al., 2019). Disrupted neural communication within and between the DMN, SN, VAN, DAN, and ECN has been documented among individuals who have sustained brain injury (He et al., 2007; Jilka et al., 2014; Churchill et al., 2021), which may slow information processing and cause lapses in attention (i.e., inattentional blindness) that perturb perception (Kenzie et al., 2017). Thus, RTI appears to offer a better index of top-down control of attention than an individual's average RT (Johnson et al., 2015).

Studies of RTI have almost exclusively defined the end of each measurement interval by a simple motor response from a seated position (e.g., keyboard tap, mouse click, button press), which requires minimal integration of visual attention and cognitive decision-making with sensorimotor processes. Although RTI may provide the best representation of inefficiency in the performance of extremely fast perceptual processes, Dispersion and Discrepancy values may nonetheless provide unique information about neural processes. Performance metrics like Speed, Acceleration, and Deceleration only have relevance in the context of goal-directed whole-body movements that closely replicate sport-related maneuvers. Combining a cognitive challenge with execution of a sufficiently complex motor activity can clearly reveal performance deficiencies that are not apparent when the respective tasks are done separately, which is believed to be due to finite neural processing resources (Leone et al., 2017). The cerebellum appears to be particularly important for efficient performance of simultaneous cognitive-motor activity, which broadens the volume of information processing that needs to be integrated among spatially separated brain areas (Wu et al., 2013). Additionally, WBRA movements presumably require complex interhemispheric interactions for coordination of bilateral muscle activation patterns (Serrien et al., 2006; Takeuchi et al., 2012; Davidson and Tremblay, 2016). Individuals differ in terms of the locations of neural circuit integration zones (Greene et al., 2020), and probably many other aspects of connectivity among brain networks as well, which makes some amount of IIV across differing assessments of interrelated performance capabilities highly likely (Schretlen et al., 2003; Holtzer et al., 2008; MacDonald et al., 2009; Roalf et al., 2016). Thus, small Dispersion and Discrepancy values may index optimal neural efficiency, whereas excessively large values may be useful clinical indicators of impaired network connectivity (Costa et al., 2019).

Traditional reductionistic study designs, combined with parametric data analyses that treat IIV as measurement error, have probably limited the ability of many prior investigations to detect subtle and heterogenous impairments that could explain the association of SRC with elevated musculoskeletal injury incidence (Quatman et al., 2009; Kenzie et al., 2017). Reductionism refers to an attempt to reduce a complex problem into components that can purportedly be studied separately for attainment of an understanding of the whole phenomenon (Quatman et al., 2009; Bittencourt et al., 2016), whereas a complex systems approach involves a search for high-order composite variables that represent the collective function of multiple interacting elements (Kenzie et al., 2017; Fonseca et al., 2020). Rather than seeking to identify isolated risk factors that may be a primary cause of subsequent injury, a complex systems approach involves identification of patterns among interacting factors that lead to the emergence of elevated injury susceptibility (Bittencourt et al., 2016). A single assessment (e.g., pre-participation physical examination) is incompatible with a complex systems approach, which requires data from multiple time points to obtain a better representation of global behavior (Fonseca et al., 2020). With regard to assessment of within-session IIV, the data derived from a single measurement session could be affected by factors that vary over time, such as motivation, fatigue, stress, emotions, or pain. Thus, a single measurement may reflect a temporary state, whereas an average of measurements obtained over multiple assessment sessions is more likely to represent a stable trait (Stawski et al., 2019a).

### Limitations and Future Directions

A major limitation of this study was the small cohort size, which was based on the available number of elite athletes who were willing to voluntarily commit to completion of the training program. A related limitation was the small number of female participants (6/16), which may have influenced our finding of no sex influence on the discriminatory power of the composite predictor variables. The lack of a sex effect could have been due to use of relative, rather than absolute, performance metrics to create Global Index values. Although WBRA Asymmetry metrics exhibited poor test-retest reliability, the corresponding performance metrics for the left and right movements from which they were derived demonstrated good to excellent reliability. The inconsistency of Asymmetry values across training sessions might be an indicator of inefficient perceptual-motor processes, which might explain the strong association of the Composite Asymmetry metric with HxSRC. Our use of the same motion analysis system for assessment and training of perceptual-motor efficiency precluded investigation of a transfer of training effect to some other ecologically valid measurement of dynamic sport performance capability, and the limited availability of the participants precluded investigation of the extent to which benefits were retained beyond the end of the training period. Future research with a motion analysis system that will provide data for each successive task repetition should compare the discriminatory power of IIV Inconsistency to that provided by Dispersion and Discrepancy calculated from the same dataset. Because a minimum of 7 measurements are recommended for assessment of IIV (MacDonald et al., 2009), lack of separate body displacement measurements for left and right movement directions limited our analysis of averaged WBRA Excursion values for the 4 task modes. The possible relevance of Excursion to visuospatial calibration between egocentric and allocentric coordinates makes it another neuromechanical performance metric that may contribute to a high-order representation of perceptual-motor efficiency (van der Ham et al., 2014; van Andel et al., 2017). A future means to acquire separate measurements for congruent and incongruent trials of the flanker test used for the DT mode might provide further insights relevant to inhibitory control.

Despite a lack of neuroimaging or electrophysiological data that directly relate our WBRA results to neural correlates within the same individuals, a strong theoretical basis exists for the premise that the Dispersion, Discrepancy, Composite Asymmetry, and Excursion Average differences we demonstrated between HxSRC and NoSRC athletes may represent high-order evidence of impaired connectivity among brain networks. Reduced SN deactivation of the DMN among individuals who have sustained traumatic brain injury has been shown to adversely affect detection of salient stimuli and integrated neural processing of visuospatial information (Jilka et al., 2014; Antonakakis et al., 2016; Churchill et al., 2021). Our interpretations of the study results are based on 3 interrelated assumptions: (1) Self-reported HxSRC greatly increases the likelihood that microstructural damage to white matter tracts has had long-term adverse effects on perceptual-motor efficiency, (2) A Global Index derived from multiple WBRA task modes, performance metrics, and measurement sessions provides a better representation of perceptual-motor efficiency than traditional group-level central tendency statistics or any single performance metric derived from a single measurement session, and (3) Change in Global Index cut points and median values between early and late phases of a training program provide evidence of improvements in perceptual-motor efficiency that are probably attributable to improved functional connectivity within and between brain networks. To the extent that these assumptions are valid, our findings may have profound implications for clinical management of SRC and training for both injury risk reduction and enhancement of sport performance capabilities. More research is clearly needed to validate our findings in larger cohorts representing different populations, and to prospectively assess a possible effect of WBRA training on the incidence of sport-related injury.

The potential long-term consequences of SRC and repetitive “subconcussive” head impacts make early detection of any impairment of brain function an important consideration for protection of an athlete's performance capabilities and health-related quality of life (Hirad et al., 2019). However, the clinically silent nature of the underlying pathophysiological process, combined with the reluctance of some athletes to report persistent SRC symptoms, may result in an unrecognized vulnerability to repetitive injury and progressive dysfunction (Brett et al., 2020; Ledreux et al., 2020). The ability to rapidly generate appropriate responses to visual cues may facilitate collision avoidance and impact preparation, thereby preventing injury (Kung et al., 2020). Multi-modal neuromuscular training has been shown to reduce the incidence of both musculoskeletal injury and SRC among rugby players (Hislop et al., 2017; Attwood et al., 2018), and there is evidence from prior studies indicating that WBRA performance can be improved through training (Serpell et al., 2011; Wilkerson et al., 2021a). Furthermore, the findings of studies that have utilized the flanker test to assess the perceptual-cognitive skills of athletes suggest that long-term training for enhancement of sport-specific skills requiring both inhibitory control and rapid responses may induce beneficial neuroplastic adaptations (Wang et al., 2017; Wylie et al., 2018). Lacking any known adverse effects, our methods for assessment and training of perceptual-motor efficiency appear to offer a novel and potentially beneficial approach to injury risk reduction and performance enhancement.

## Conclusions

Intra-individual variability represented as Dispersion among standardized neuromechanical performance metrics, as well as Discrepancy between maximum and minimum standardized scores, provided exceptionally good discrimination between elite athletes who either affirmed or denied a history of sport-related concussion. Composite Asymmetry and Excursion 4-Mode Average also provided good discrimination, both of which offer the advantage of easy calculation from performance metrics provided by the system used for assessment and training of perceptual-motor efficiency. Reaction time, deceleration, and precise control of whole-body movements directed to rapid deactivation of virtual reality targets appear to collectively differentiate elite athletes on the basis of whether or not a sport-related concussion has been sustained at any point in the past. Composite Asymmetry ≥0.15 and Excursion 4-Mode Average ≥ 7 m (or T-score Average <50) appear to be reasonable qualitative approximations for clinical identification of suboptimal perceptual-motor performance. Although more research is needed to document the potential value of whole-body perceptual-motor training for injury risk reduction, athlete participation in a relatively small number of brief training sessions resulted in substantial performance improvements. Our findings clearly support the potential for improved prevention and management of sport-related injuries through a complex systems approach that identifies high-order composite variables representing the collective function of multiple interacting perceptual and motor processes.

## Data Availability Statement

The raw data supporting the conclusions of this article will be made available by the authors, without undue reservation.

## Ethics Statement

The studies involving human participants were reviewed and approved by Institutional Review Board of the University of Tennessee at Chattanooga. The patients/participants provided their written informed consent to participate in this study.

## Author Contributions

All authors made significant contributions to the conceptualization, design, and implementation of the study. The manuscript draft was written by GW, with the content of the final draft revised in response by the co-authors (DN and TP) and reviewers. All components of the data analysis were done by GW and reviewed by the co-authors to ensure that the results were derived from accurate data collected by TP under the direct supervision of DN.

## Conflict of Interest

Whole-body reactive agility data were acquired from equipment loaned to the United States Olympic and Paralympic Committee by Traq Global Ltd (Westlake, OH). The authors declare that the research was conducted in the absence of any commercial or financial relationships that could be construed as a potential conflict of interest.

## Publisher's Note

All claims expressed in this article are solely those of the authors and do not necessarily represent those of their affiliated organizations, or those of the publisher, the editors and the reviewers. Any product that may be evaluated in this article, or claim that may be made by its manufacturer, is not guaranteed or endorsed by the publisher.

## Supplementary Material

Supplementary Video can be downloaded through this link: https://youtu.be/RtHrslYOxtQ
