# Supplementary figures and images for: A Novel Approach to Assessment of Perceptual-Motor Efficiency and Training-Induced Improvement in the Performance Capabilities of Elite Athletes
Source: Front Sports Act Living. 2021 Oct 1;3:729729. doi: 10.3389/fspor.2021.729729 (PMC8517233; doi:10.3389/fspor.2021.729729)

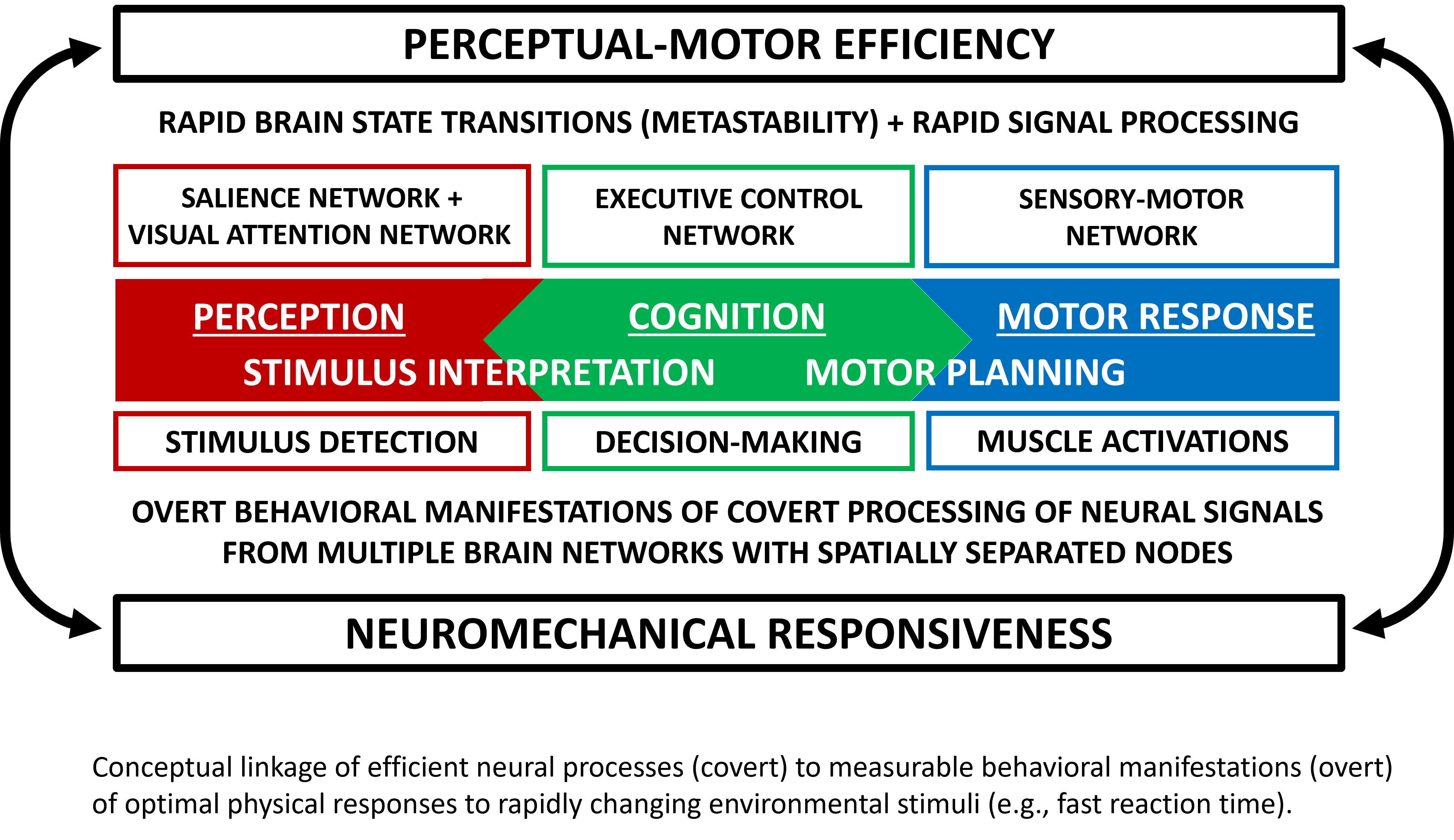

Supplement: Supplementary file 1 [file Image_1.png]
